# Repurposing dimethyl fumarate as an antiepileptogenic and disease-modifying treatment for drug-resistant epilepsy

**DOI:** 10.1186/s12967-023-04695-2

**Published:** 2023-11-08

**Authors:** Sereen Sandouka, Prince Kumar Singh, Aseel Saadi, Rhoda Olowe Taiwo, Yara Sheeni, Taige Zhang, Larin Deeb, Michelle Guignet, Steve H. White, Tawfeeq Shekh-Ahmad

**Affiliations:** 1 Faculty of Medicine, The School of Pharmacy, The Institute for Drug Research, The Hebrew University of Jerusalem, Jerusalem, Israel; 2 Department of Pharmacy, Center for Epilepsy Drug Discovery, University of Washington, Seattle, WA USA

**Keywords:** Dimethyl fumarate, Drug-resistant epilepsy, Status epilepticus, Temporal lobe epilepsy, Nrf2

## Abstract

**Background:**

Epilepsy affects over 65 million people worldwide and significantly burdens patients, caregivers, and society. Drug-resistant epilepsy occurs in approximately 30% of patients and growing evidence indicates that oxidative stress contributes to the development of such epilepsies. Activation of the Nrf2 pathway, which is involved in cellular defense, offers a potential strategy for reducing oxidative stress and epilepsy treatment. Dimethyl fumarate (DMF), an Nrf2 activator, exhibits antioxidant and anti-inflammatory effects and is used to treat multiple sclerosis.

**Methods:**

The expression of Nrf2 and its related genes in vehicle or DMF treated rats were determined via RT-PCR and Western blot analysis. Neuronal cell death was evaluated by immunohistochemical staining. The effects of DMF in preventing the onset of epilepsy and modifying the disease were investigated in the kainic acid-induced status epilepticus model of temporal lobe epilepsy in rats.

The open field, elevated plus maze and T-Maze spontaneous alteration tests were used for behavioral assessments.

**Results:**

We demonstrate that administration of DMF following status epilepticus increased Nrf2 activity, attenuated status epilepticus-induced neuronal cell death, and decreased seizure frequency and the total number of seizures compared to vehicle-treated animals. Moreover, DMF treatment reversed epilepsy-induced behavioral deficits in the treated rats.

Moreover, DMF treatment even when initiated well after the diagnosis of epilepsy, reduced symptomatic seizures long after the drug was eliminated from the body.

**Conclusions:**

Taken together, these findings suggest that DMF, through the activation of Nrf2, has the potential to serve as a therapeutic target for preventing epileptogenesis and modifying epilepsy.

**Supplementary Information:**

The online version contains supplementary material available at 10.1186/s12967-023-04695-2.

## Background

Over 65 million people worldwide suffer from epilepsy, one of the most prevalent and debilitating neurological diseases [1]. Epilepsy is responsible for a substantial share of the global disease burden [2], morbidity, and mortality [3, 4]. Presently, notwithstanding the availability of many FDA-approved antiseizure medications (ASMs) over the past several decades with varying modes of action, the overall outcomes in newly diagnosed epilepsy patients have not improved [5–7]. Almost one-third of adults with epilepsy continue to experience seizures, and their disease remains uncontrolled [8, 9]. However, in responsive cases, ASMs can only symptomatically suppress seizures but do not modify the underlying pathophysiology of epilepsy [10]. Therefore, there is a clear need for new treatment strategies with effective anti-epileptogenic therapies to prevent, modify, or reverse the cellular and molecular mechanisms of epileptogenesis [11, 12]. Moreover, ASM resistance is of particular concern in patients with temporal lobe epilepsy (TLE), as is progressive hippocampal atrophy and subfield neuronal loss [13–15]. Strategies to prevent the emergence of drug-resistant epilepsy (DRE) would be of significant value to patients and their healthcare providers.

In the last few decades, growing attention has been focused on the mechanisms by which endogenous cytoprotective defense processes combat the pathological mechanisms underlying many neurodegenerative diseases including epilepsy [16]. Unregulated production of reactive oxygen species (ROS) and oxidative stress are among the pathological features [17, 18] that might contribute to oxidative stress-induced neuronal cell death and disease pathogenesis [19, 20]. The pathophysiological features associated with an imbalance between harmful ROS and protective antioxidant defenses are primarily controlled by endogenous cytoprotective pleiotropic transcription factor nuclear factor-erythroid 2-related factor 2 (Nrf2) [21]. Therefore, the functionality of the Nrf2-kelch-like erythroid-cell-derived protein with the CNC homology (ECH)-associated protein1 (Nrf2-Keap1) axis is necessary for protection against diseases that involve oxidative stress and ROS as a pathophysiological mechanism. Nrf2 plays a significant role in cellular cytoprotective responses by regulating the expression of several antioxidant and cytoprotective genes [22]. Under basal conditions, Nrf2 is sequestered in the cytoplasm by the repressor protein Keap1 [23]. However, under cellular stress (i.e., oxidative stress), it translocates to the nucleus, where it utilizes its transcriptional activity to induce the production of many defense proteins, including heme oxygenase 1 (HO-1) and NAD(P)H: quinone oxidoreductase-1 (NQO1) [22, 24, 25]. Kraft et al*.,* reported that Nrf2 knockout mice were more sensitive to kainate toxicity, as evidenced by elevated seizure severity, duration, hippocampal neuronal damage, and mortality [26]. Wang et al*.,* also found that kindled seizures in rats caused oxidative stress. Moreover, it was demonstrated that the expression of Nrf2 and its downstream genes HO-1 and NQO1, at the protein and gene levels, significantly increased in the hippocampus following seizure induction, suggesting that the Nrf2 signaling pathway was activated in the hippocampus after a seizure [27].

DMF was approved by the FDA for the treatment of multiple sclerosis in 2013, and its application in the treatment of various tumors, inflammatory bowel disease, and intracranial hemorrhage has been reported in subsequent studies [28, 29]. Although its exact mechanism of action is unknown, orally administered DMF is thought to exert its therapeutic (e.g., neuroprotective, anti-inflammatory) effects via activation of the Nrf2 antioxidant response pathway [30, 31]. It has also been shown that Monomethylfumarate, the primary active metabolite of DMF, induces Nrf2 activation in vitro in cells transfected with rat Keap-1, a protein that facilitates Nrf2 proteolysis, resulting in translocation of Nrf2 to the nucleus and subsequent induction of antioxidant response genes [32].

In the present study, we demonstrated that DMF, administered shortly after SE and before the onset of spontaneous recurring seizures, elevated Nrf2 activity and attenuated SE-induced neuronal cell death in the CA1 and CA3 regions of the hippocampus of treated animals. Moreover, DMF treatment significantly decreased the total seizure burden compared to vehicle-treated animals and prevented epilepsy development in 3/10 animals. Furthermore, we found that 1 week of DMF treatment in rats with established epilepsy significantly decreased seizure frequency and cumulative number of seizures compared to vehicle (Veh) rats. Interestingly, this effect persisted for 3 weeks after DMF treatment, suggesting that Nrf2 activation may be disease-modifying, even when the presence of the circulating drug has been eliminated.

Together, these results suggest that DMF, an FDA-approved drug, may be repurposed as a safe and effective therapy, offering a potential first-in-class disease-modifying agent in newly diagnosed epilepsy and therapy for people at risk of developing epilepsy following a brain insult, as well as for patients with established epilepsy.

## Methods

### Experimental epilepsy models

#### Animals

**Scheme 1:**
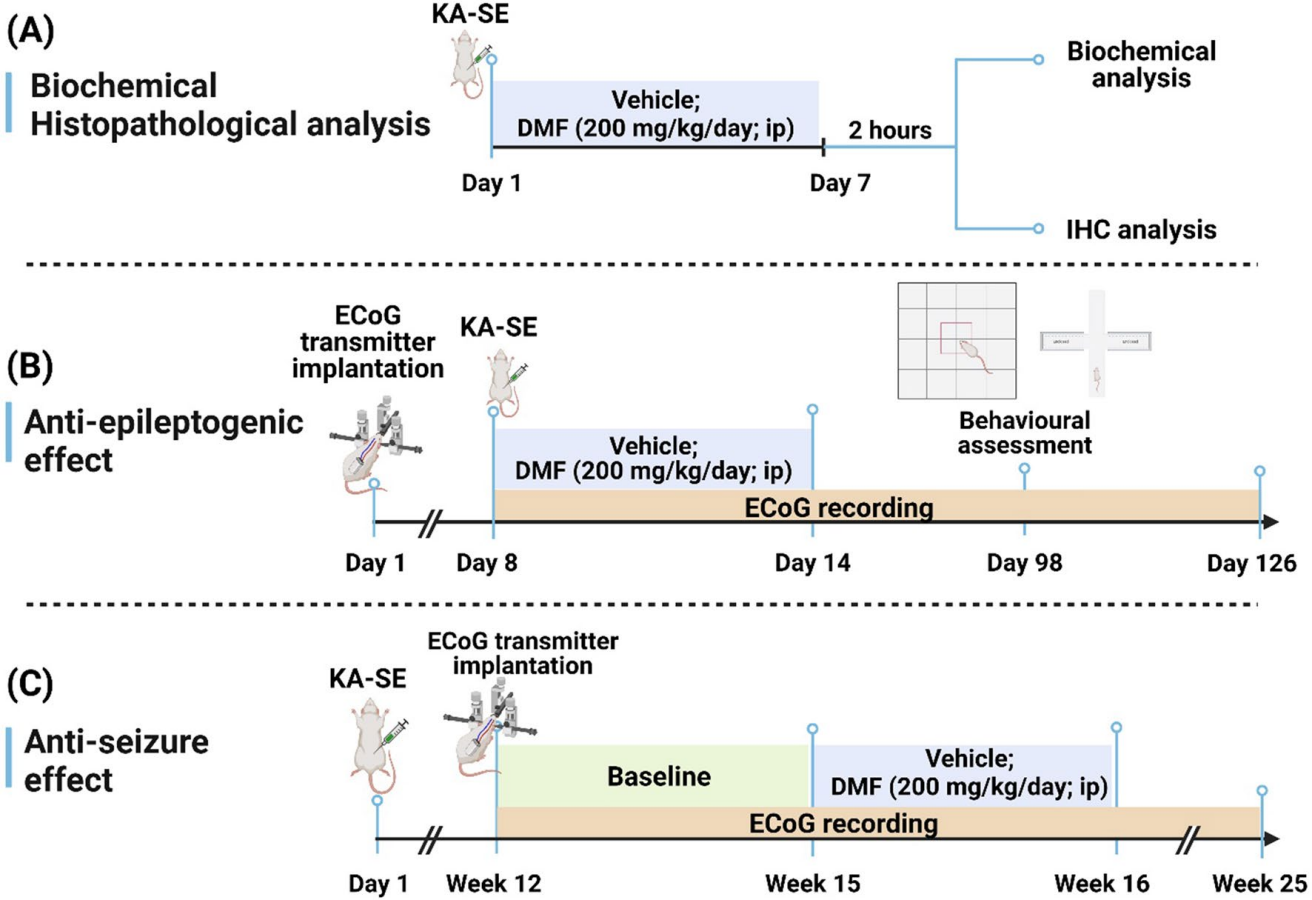
Schematic overview of the experimental design. SD rats were subjected to kainic acid-induced status epilepticus (KA-SE) followed by treatment with either vehicle or DMF (200 mg/kg/day, ip). Treatments were initiated within 10 min after diazepam injection (for termination of SE), followed by once daily administration for an additional 6 days. The vehicle group received an equivalent volume and number of injections, similar to those in the DMF group. **A** For biochemical and histological analyses, rats were sacrificed 2 h after the last dose (7 days after SE). **B** To evaluate the anti-epileptogenic effect, ECoG devices were implanted into rat brains 1 week before KA-SE, followed by treatment with vehicle or DMF for one week after SE. Video-ECoG monitoring was conducted for up–16-18 weeks after SE. Behavioral testing was performed at week 13 after SE (the time of chronic epilepsy). At the end of the video-ECoG recording, the rat brains were harvested for histological analysis. **C** To test the anti-seizure effect of DMF, we induced KA-SE in rats, then 10–12 weeks later, we implanted ECoG devices into rats and conducted video-ECoG monitoring. We performed baseline video-ECoG monitoring for 3 weeks, followed by treatment with the vehicle or DMF for 1 week. Rats were sacrificed at the end of the video-ECoG monitoring period

The study was conducted on male Sprague Dawley (SD) rats (weighing 150–200 g) obtained from the Harlan strain of Hebrew University of Jerusalem, Israel. Animals were individually housed with free access to food and water under standard conditions (23 ± 1 °C, 50–60% humidity; 12-h light/dark cycle). Standard rodent chow was provided ad libitum. The total number of animals was 82 rats:12 were used as sham animals, and 70 received kainic acid (KA), of which 62 survived SE and surgery and were included in the study (Scheme 1). All procedures described herein were conducted according to the Association for Assessment and Accreditation of Laboratory Animal Care International and approved by the Institutional Animal Care and Use Committee of the Hebrew University of Jerusalem (Approval No.: MD-20–16254-5).

#### The KA-induced SE epilepsy model and treatment

To induce SE, rats were injected with KA according to a previously described protocol [33, 34]. Rats were injected intraperitoneally with KA (Hello Bio) dissolved in sterile 0.9% saline (5 mg/ml) at a dose of 5 mg/kg hourly, followed by continuous monitoring of the development of convulsive motor seizures. KA injections were continued until repeated class IV or V seizures were evoked (scored according to a modified Racine’s scale) [35]. Rats were included in the study only when they reached class V seizures (i.e., excessive rearing with concomitant forelimb clonus and falling) or repeated stage IV seizures within one hour. To reduce mortality, SE was terminated in all animals after 2 h by intraperitoneal administration of diazepam (10 mg/kg). Animals were randomized into treatment groups at the beginning of the injection scheme to prevent any bias in the duration of SE or the total dose of KA. Baseline SE characteristics of the Veh and DMF groups are shown in Additional file 1: Fig. S1.

#### Surgery: implantation of wireless electrocorticography (ECoG) transmitters

Before surgery, rats were anesthetized with isoflurane (3%) and shaved at the site of the planned incision for electrode insertion. The animals were then placed in a stereotaxic frame (Kopf, CA, USA) under isoflurane anesthesia (1.5–2%). Rats received buprenorphine (0.2 mg/kg; SC) and Metacam (1 mg/kg; SC) for pain relief. The head was sterilized with a povidone-iodine antiseptic solution and the skull was exposed using a single rostrocaudal scalp incision. Burr holes were drilled into the skull using a high-speed stereotaxic drill at the following coordinates (relative to bregma), recording electrode: + 2.5 mm ML, − 3.0 mm AP; reference electrode: − 2.0 mm ML, − 6.0 mm AP. The electrode wires were inserted into the burr holes and secured in position with stainless-steel screws. The ECoG transmitter (A3028E; Open-Source Instruments, OSI) was placed in a subcutaneous cavity formed over the left flank with a single skin incision.

The incision was sealed with dental cement at the end of the surgical procedure. Warm Ringer's solution (3–5 mL) and amoxicillin (100 mg/kg Betamox LA, Norbrook, UK) were administered to individual rats before returning them to their home cages for recovery.

Rats were housed individually in Faraday cages and allowed to recover for seven days before commencing the experiment. During the time that KA was being injected, rats were subjected to ECoG recording (to monitor for the development of SE). Rats were subsequently subjected to continuous video-ECoG recording for up to 18 weeks following the SE to monitor for the efficacy of treatment on epilepsy development.

#### Video-electrocorticography analysis

Continuous electrocorticography (ECoG) was acquired from a subdural electrode 24/7 using Neuroarchiver software (OSI) and analyzed as previously reported [34, 36].

In brief, the ECoG data were segmented into 4-s epochs, and six metrics were evaluated (power, intermittency, coastline, coherence, asymmetry, and power between 12 and 30 Hz). Each metric was mapped onto an interval of 0–1 and then compared to a user-generated seizure library containing seizures from at least three different animals (https://www.opensourceinstruments.com/Electronics/A3018/Event_Detection.html).

Epochs were considered seizure-like events if their Euclidean distance was less than 0.2 of a validated seizure epoch. All electrographic seizures were validated by visual inspection by a researcher who was blinded to treatment.

A selection of seizures was also characterized by a researcher blinded to treatment by video monitoring obtained using digital time-locked CCTV cameras (Microseven) placed outside the cages.

### Drug administration

Dimethyl fumarate (DMF; Sigma) was dissolved in a mixture of methylcellulose 0.5% (80%) and PEG 400 (20%) (Sigma-Aldrich) at a concentration of (50 mg/ml) and administered intraperitoneally once daily to rats at a total dose of 200 mg/kg/day for 1 week. Control animals were treated with an equivalent volume and number of injections of the vehicle as the DMF-treated animals. For biochemical and antiepileptogenic experiments, rats were treated with either DMF or vehicle for 1 week after SE. For the chronic epilepsy experiment, rats with spontaneous recurrent seizures (SRS) were treated with either DMF or vehicle for 1 week after a 3-week baseline recording period.

### Behavioral tests

Anxiety-like behavior was evaluated in rats using Elevated plus maze (EPM) and open-field (OF) tests. The spontaneous alternation T-Maze was used to assess spatial learning and decision-making processes.

#### Elevated plus maze

The EPM consists of two opposing open arms (50 × 12 cm) and two enclosed arms (50 × 12 cm) extending from a central platform (12 × 12 cm), elevated 50 cm above the floor. The rats were habituated to the testing room for 1 h before testing. On the day before the test, each animal was placed in the center of the maze and allowed to explore for approximately 10 min. After the exploration phase, the rat was placed at the intersection of the open and closed arms of each maze to start the 5-min test (a beam break in this area triggered the start of the test). During the 5 min of the test, the animal may spend time either in a closed safe area (closed arms) or in an open area (open arms). The movements of the animals were recorded using an overhead camera. A semi-automatic script written in DeepLabCut™ (https://github.com/DeepLabCut) was used to analyze the video recordings to assess the entries and time spent in the open arms of the maze. The maze was cleaned with a 70% ethanol solution between each animal.

#### Open field

The OF maze was made of a plexiglass box with dimensions of 100 × 100 × 40 cm. The animal was introduced into the OF arena and allowed to move freely for 10 min while being recorded using an overhead camera. Footage was then analyzed using a semi-automatic script written in DeepLabCut™ (https://github.com/DeepLabCut). During the 5 min of the test period, the total distance moved by the animal and the time spent in the center of the arena (50 cm × 50 cm) were tracked and measured. The maze was cleaned with 70% ethanol between each animal.

#### Spontaneous alternation T-maze

The T-maze apparatus employed in this study consisted of a symmetrical T-shaped structure comprising a central stem and two perpendicular arms (50 × 12 cm) extending from the stem to form a T-shape. In each trial, the rat was initially placed in the start box and allowed to navigate through the stem of the maze, where it could choose either of the maze arms. To be considered as having entered a specific arm, the rat had to enter that arm with all limbs. Once the rat entered a goal arm, a guillotine door was closed for 30 s. The rat was returned to the starting box to begin the next trial. The intertrial interval was 1 min. The arms selected by the rats were recorded for each trial. After completion of the experimental period, consisting of six trials for each rat, the total number of alternations was recorded. The percentage bias for each rat was calculated using the following formula: (total number of correct alternations/6) × 100. All the experiments were conducted by an experimenter blinded to the treatment group.

### Immunohistochemistry

For immunohistochemistry studies, rats were euthanized on day 7 post-SE, 2 h after the final dose of DMF or VEH, with intraperitoneal administration of ketamine and xylazine (100 mg/kg and 10 mg/kg, respectively). The rats were then perfused with heparinized ice-cold PBS, followed by transcardial perfusion with 4% paraformaldehyde (PFA; Sigma) in PBS (pH 7.4). The brains were removed, fixed overnight with 4% paraformaldehyde solution (PFA, Sigma) in PBS (pH 7.4), and cryoprotected in sucrose phosphate buffer (10%, 20%, and 30% solutions for 24 h each) at 4 $^\circ$C. After freeze-mounting, brain samples were immersed in O.C.T compound (Scigen) and stored at − 80 $^\circ$C. For immunohistochemical studies, coronal sections of 20 μm selected from the hippocampus were cut in a cryostat (Leica CM1950) at − 20 °C, fixed on poly L-lysine coated slides (Thermo Fisher Scientific), and left to dry for 2 h. Each slide contained 2–3 sections per animal. The primary antibody was added after washing thrice with PBS for 10 min each. Washed sections were incubated overnight at 4 °C with rabbit primary antibody against the neuronal marker NeuN (1:500, ab177487, Abcam, Cambridge, UK) in a solution of PBS, 0.1% Triton X-100 and BSA 1%. Following three washes with PBS for 10 min each, the sections were incubated with Alexa Fluor^®^ 488 goat anti-rabbit secondary antibody (1:500; ab150081, Abcam, Cambridge, UK) for 2 h at room temperature in the dark. The sections were washed three times with PBS for 10 min each and mounted with Vectashield and 4′, 6-diamidino-2-phenylindole (DAPI) mounting medium (Vector Labs). Images were obtained at a resolution of 1024 × 1024 using a Nikon confocal A1R microscope with 20X and 40X objectives. Images were acquired at 405 nm excitation wavelength and 455 nm emission wavelength for DAPI and at an excitation of 495 nm and emission of 519 nm for NeuN. Image analysis was performed using ImageJ software with a manual cell counting image-based tool, and the investigators were blinded to the treatment. The results are expressed as cell density, that is, the average number of NeuN + cells per mm^2^, for 2-brain sections per rat in 2–3 images of ROIs of CA1 and CA3 subfields of the hippocampus.

### Western blotting

Rats were sacrificed 2 h after the final dose of DMF or vehicle at 7 days post-SE. The rats were euthanized by intraperitoneal administration of ketamine and xylazine (100 mg/kg and 10 mg/kg, respectively). Frozen brains were immediately extracted and stored at − 80 °C until they were thawed on ice, resuspended in RIPA buffer (Thermo Fischer), homogenized, and incubated at 4 °C for 2 h under constant rotation. Protein samples were centrifuged and prepared, and 25 µg of each sample was transferred onto nitrocellulose membranes (Bio-Rad, Hercules, CA, USA). Nonspecific binding sites on the membrane were blocked with SuperBlock buffer in TBS containing 0.1% Tween-20, 0.05% Tris-Chloride, and 0.03% 5 M NaCl (TBS-T) for 1 h at 22–24 °C. Membranes were subsequently incubated overnight at 4 °C with primary anti-NFE2L2 antibody (1:5000, 16,396–1-AP, Proteintech), anti-NQO1 antibody (1:1000, ab34173), and β actin (1:2000, ab8226). The preparative membranes were probed with appropriate secondary antibodies conjugated to HRP. Immunological complexes were visualized using electrochemiluminescence (ECL, Bio-Rad). Densitometry analysis was performed using Image Lab software (5.1, Bio-Rad, Hercules, CA, USA). Data were normalized to the loading control (β-actin).

### Quantitative real-time polymerase chain reaction

Immediately following euthanasia, total RNA was extracted from brain tissue using TRI Reagent (Sigma-Aldrich, St. Louis, MO, USA) and kept at − 80 °C following animal sacrifice. One microgram of RNA was used as Complementary DNA (cDNA) using the GoScript™ Reverse Transcription System (Promega, Madison, WI, USA) with an oligo-dT15 primer. Real-time PCR was carried out in a final volume of 15 μL (7.5 μL of SYBR Green, 3 μL of primers (500 nM each), 1.5 μL of DEPC water, and 3 μL of cDNA template was added to the mixture) with SYBR Green (PerfeCTa SYBR Green FastMix, Quantabio) and monitored by CFX Connect Real-Time PCR Detection System (Bio-Rad). The following cycling parameters were used: 10 min at 95 °C, followed by 40 cycles of denaturation at 95 °C for 5 s, and 60 °C for 15 s. Finally, 5 s at 65 °C, and 30 s at 95 °C. Primer sequences are reported in Table 1. Each RT-PCR was performed in duplicate. The level of target mRNA was quantified relative to the housekeeping gene (GAPDH) using the ΔΔCT method [37]. GAPDH expression was not significantly different between treatments.

**Table 1:** Primers sequences used for RT-PCR

Name	Accession number	Forward	Reverse
Nrf2	NM_031789	GCAACTCCAGAAGGAACAGG	GGAATGGCTCTCTGCCAAAAGC
GAPDH	NM_017008	GACATGCCGCCTGGAGAAAC	AGCCCAGGATGCCCTTTAGT
NQO1	NM_017000	GTTTGCCTGGCTTGCTTTCA	ACAGCCGTGGCAGAACTATC
HO-1	NM_012580	ACAGGGTGACAGAAGAGGCTAA	CTGTGAGGGACTCTGGTCTTTG
GCLC-1	NM_012815	GAGTAGAGTTCCGACCAAT	GCTCCTGTGCCACTTTCA

Nrf2, nuclear factor E2-related factor 2; GAPDH, glyceraldehyde-3-phosphate dehydrogenase

NQO1 NAD(P)H: quinone oxidoreductase-1 gene, HO-1 haem oxygenase 1 gene, and GCLC1 glutamate-cysteine ligase catalytic subunit

### Statistical analysis

Data are expressed as mean ± standard error of the mean (SEM). Statistical analyses were performed using the GraphPad Prism 9.5.0 software. Data were analyzed using ordinary one-way analysis of variance (ANOVA) followed by the Tukey post-hoc test or generalized log-linear mixed model, followed by the Sidak posthoc test.

Sample sizes were chosen based on our previous calculations of experimental variability. The number of animals used is described in the corresponding figure legend.

The statistical significance threshold was determined as α error with a value of P < 0.05.

## Results

### DMF increases Nrf2 expression in the cortex and hippocampus following KA-SE

We first determined the effect of DMF on mRNA and protein levels of Nrf2 in the brain following kainic acid-induced status epilepticus (KA-SE). After SE induction, the rats were treated with either vehicle or DMF for 7 days and then sacrificed 2 h after the final dose. The mRNA and protein levels of Nrf2 were examined in the cortex and hippocampus.

**Fig. 1:**
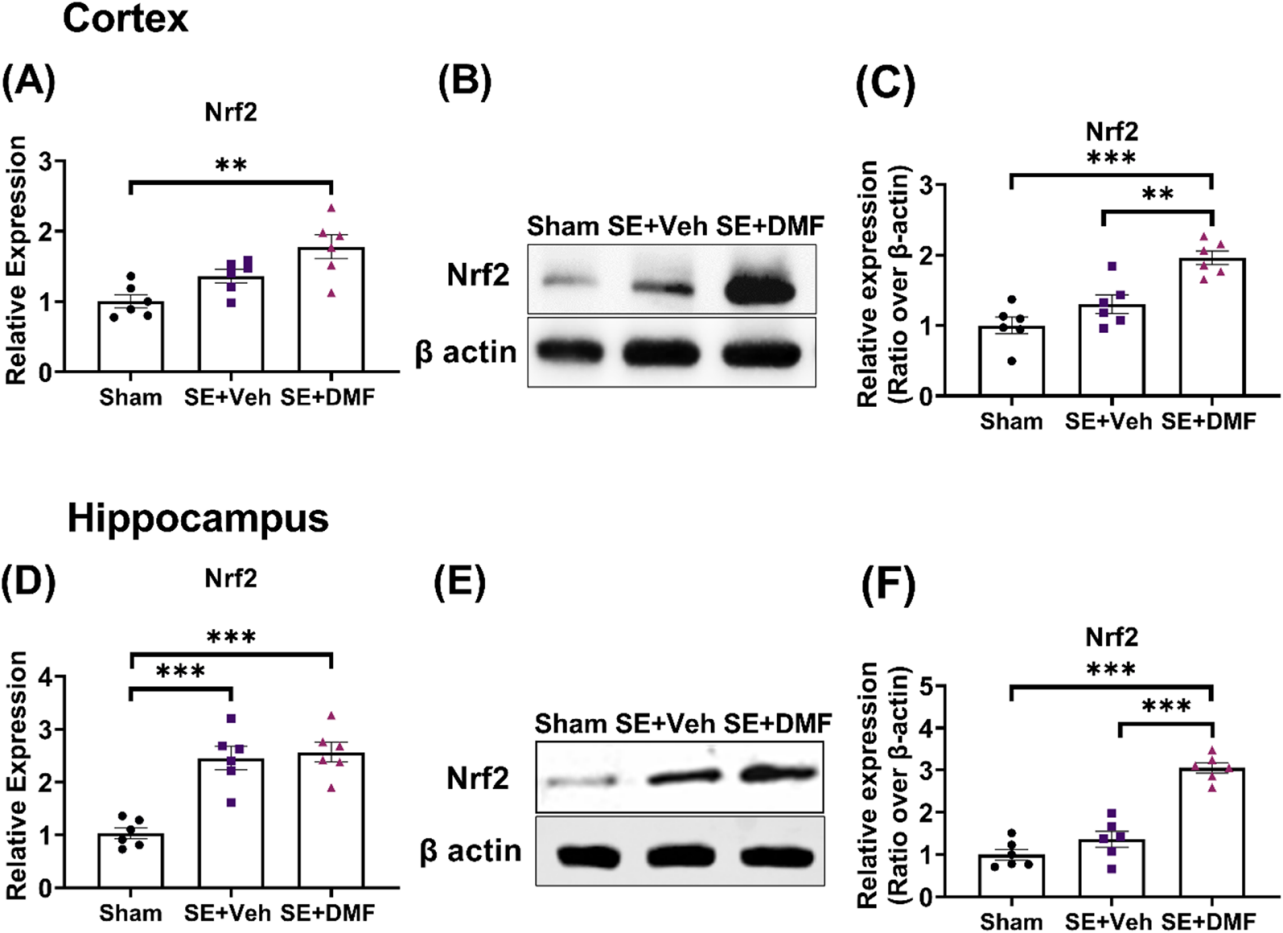
DMF increased Nrf2 expression in the cortex and hippocampus after SE. **A**, **D** Relative gene expression of Nrf2 measured by quantitative Reverse transcription-polymerase chain reaction (RT-PCR) in the cortex (**A**) and the hippocampus (**D**) of control rats (n = 6), and rats after kainic acid-induced SE (KA-SE) treated with either vehicle (n = 6) or DMF (ip, 200 mg/kg/day, 7 days, n = 6). **B**, **E** Representative western blot images of Nrf2 in the cortex (**B**) and the hippocampus (**E**). **C**, **F** Quantification of western blotting results for Nrf2 in the cortex (**C**; n = 6) and hippocampus (**F**; n = 6). Gene expression was normalized to GAPDH expression and is presented as fold-change compared to the level in sham rats. Protein levels were expressed as relative protein expression normalized to β-actin. Data are reported as the mean ± SEM. **p < 0.01 and ***p < 0.001 after one-way ANOVA followed by Tukey’s posthoc test

In agreement with our previously published study [38], a positive non-significant trend to increase Nrf2 mRNA level was observed in the cortex (Fig. 1A), and significantly increased in the hippocampus 1 week after KA-SE (Fig. 1D). DMF treatment was associated with a significant increase in Nrf2 protein levels in the cortex (Fig. 1B, C), and a higher increase in the hippocampus (Fig. 1E, F).

### DMF increases Nrf2 downstream genes in the cortex and hippocampus following KA-SE

**Fig. 2:**
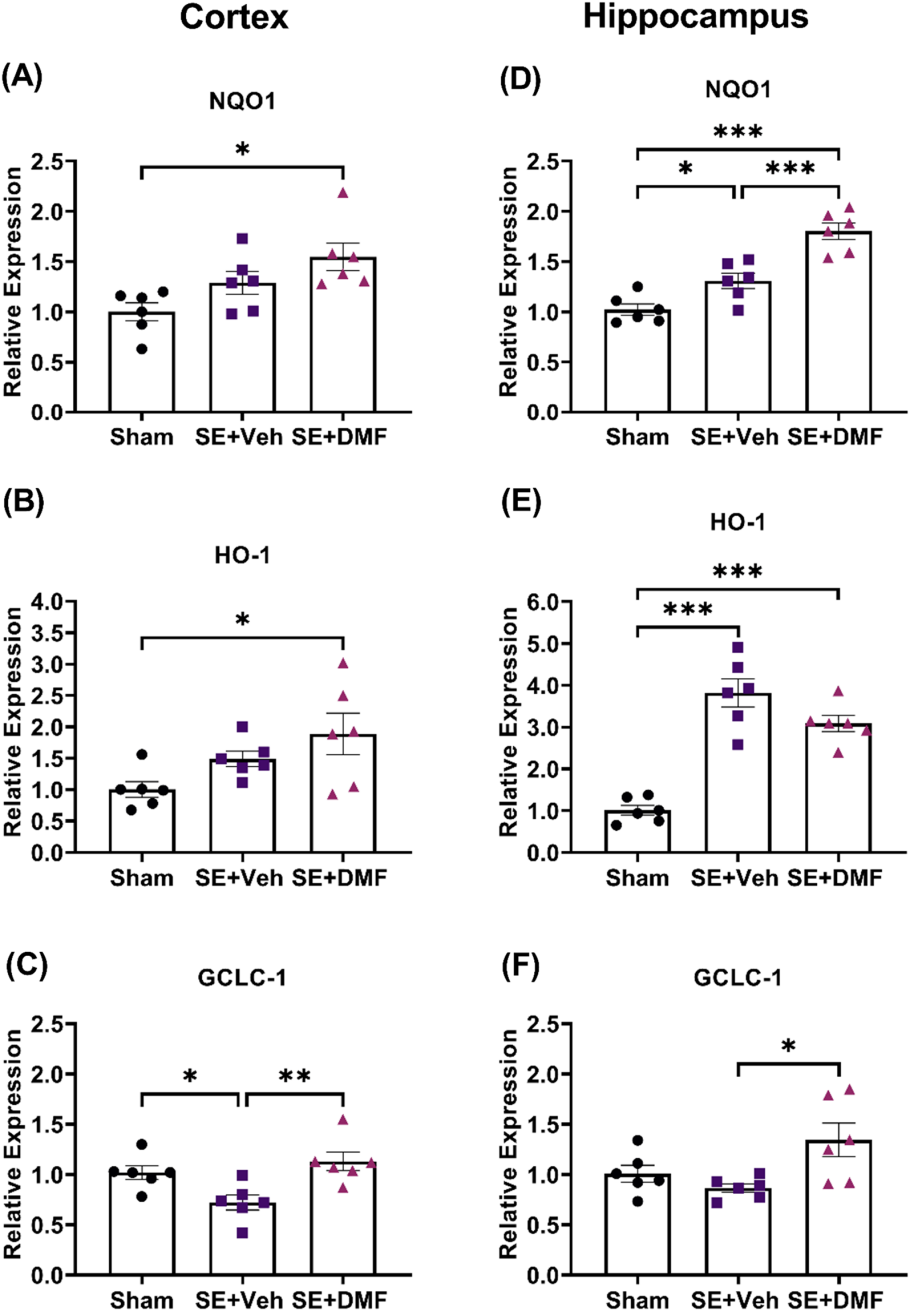
DMF increases the mRNA expression of Nrf2 downstream genes after KA-SE. Relative gene expression of Nrf2 downstream genes NQO1, HO-1, and GCLC-1, in the cortex (**A**–**C**) and the hippocampus (**D**–**F**) of control rats (n = 6), and rats after kainic acid-induced SE (KA-SE) treated with either vehicle (n = 6) or DMF (ip 200 mg/kg/day, 7 days, n = 6). Gene expression was normalized to GAPDH expression and is presented as fold-change compared to the level in sham rats. Data are reported as mean ± SEM. *p < 0.05; **p < 0.01; ***p < 0.001 after one-way ANOVA followed by Tukey’s posthoc test

The functional effect of DMF on the activation of the Nrf2 pathway was evaluated. We measured the mRNA and protein levels of the downstream genes of Nrf2, NQO1, HO-1, and GCLC-1. We found that NQO1 and HO-1 mRNA levels followed a similar regional increase as that observed for Nrf2 in the cortex (Fig. 2A, B; NQO1, Sham vs. SE + DMF: p = 0.0113; HO-1, Sham vs. SE + DMF: p = 0.0283), and the hippocampus (Fig. 2D, E; NQO1, Sham vs. SE + DMF: p < 0.0001; HO-1, Sham vs. SE + DMF: p < 0.0001).

Importantly, the protein levels of NQO1 and HO-1 also increased in both cortex (Additional file 1: Fig. S2), and the hippocampus (Additional file 1: Fig. S3).

Interestingly, the GCLC-1 mRNA level in the cortex significantly decreased following SE compared to sham animals (p = 0.0474), and treatment with DMF prevented this decrease (Fig. 2C; Sham vs. SE + DMF, p = 0.5897). Moreover, the mRNA levels of GCLC-1 in the hippocampus were not reduced following SE (p = 0.6370). However, DMF treatment increased the expression of both mRNA (Fig. 2F; SE + Veh vs. SE + DMF, p = 0.0201), and protein levels (Additional file 1: Fig. S2, S3).

### DMF decreases SE-induced neuronal cell death

The induction of KA-SE has been previously reported to result in notable hippocampal neuronal damage [39–41]. We previously reported that the KA-SE model caused significant neuronal loss in the CA1 and CA3 subfields of the hippocampus 1 week after SE [24, 34].

**Fig. 3:**
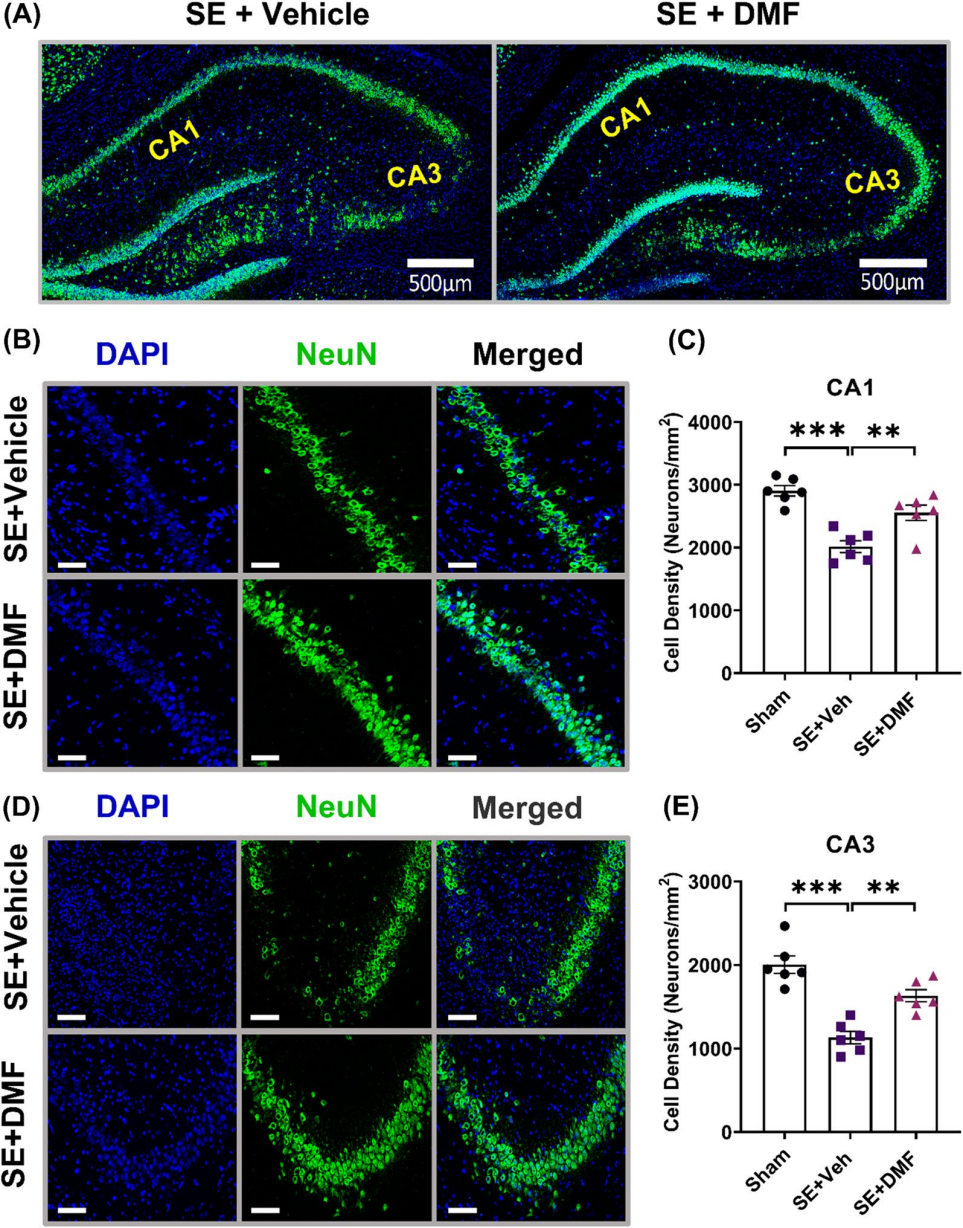
DMF prevents neuronal cell loss in the hippocampus following SE. **A** Representative images of the hippocampi of rats subjected to KA-SE and treated with vehicle (n = 6, left) or DMF (200 mg/kg/day over 7 days, n = 6, right). CA1 and CA3 (highlighted in yellow) regions were analyzed for neuronal density 1 week following KA-SE onset. Scale bar = 500 µm. **B**, **D** Representative images (zoom view) of the CA1 region (**B**) and CA3 (**D**) of animals, as in (**A**), illustrating the staining of neurons (NeuN, green). The nuclei were stained with DAPI (blue). Scale bars = 50 μm. **C**, **E** Quantification of the number of NeuN cells per 1 mm^2^ of tissue in CA1 (**C**) and CA3 (**E**). Data are expressed as the mean ± SEM. **P < 0.01 and ***P < 0.001 after one-way ANOVA followed by Tukey’s post hoc test

In light of these findings, we measured neuronal cell densities in the CA1 and CA3 subfields of the dorsal hippocampus 1 week after SE in rats treated with vehicle or DMF (Fig. 3A). In agreement with previous findings, our results indicated that SE caused a significant loss of neurons in the CA1 and CA3 regions 1-week post-SE, as evidenced by the decrease in cell density (Fig. 3B–E). Treatment with DMF for one week after SE significantly ameliorated neuronal cell death (Fig. 3B–E; CA1, SE + Veh vs. SE + DMF: p = 0.0052; CA3, SE + Veh vs. SE + DMF: p = 0.0023).

### DMF suppresses the development of spontaneous recurrent seizures following SE

**Fig. 4:**
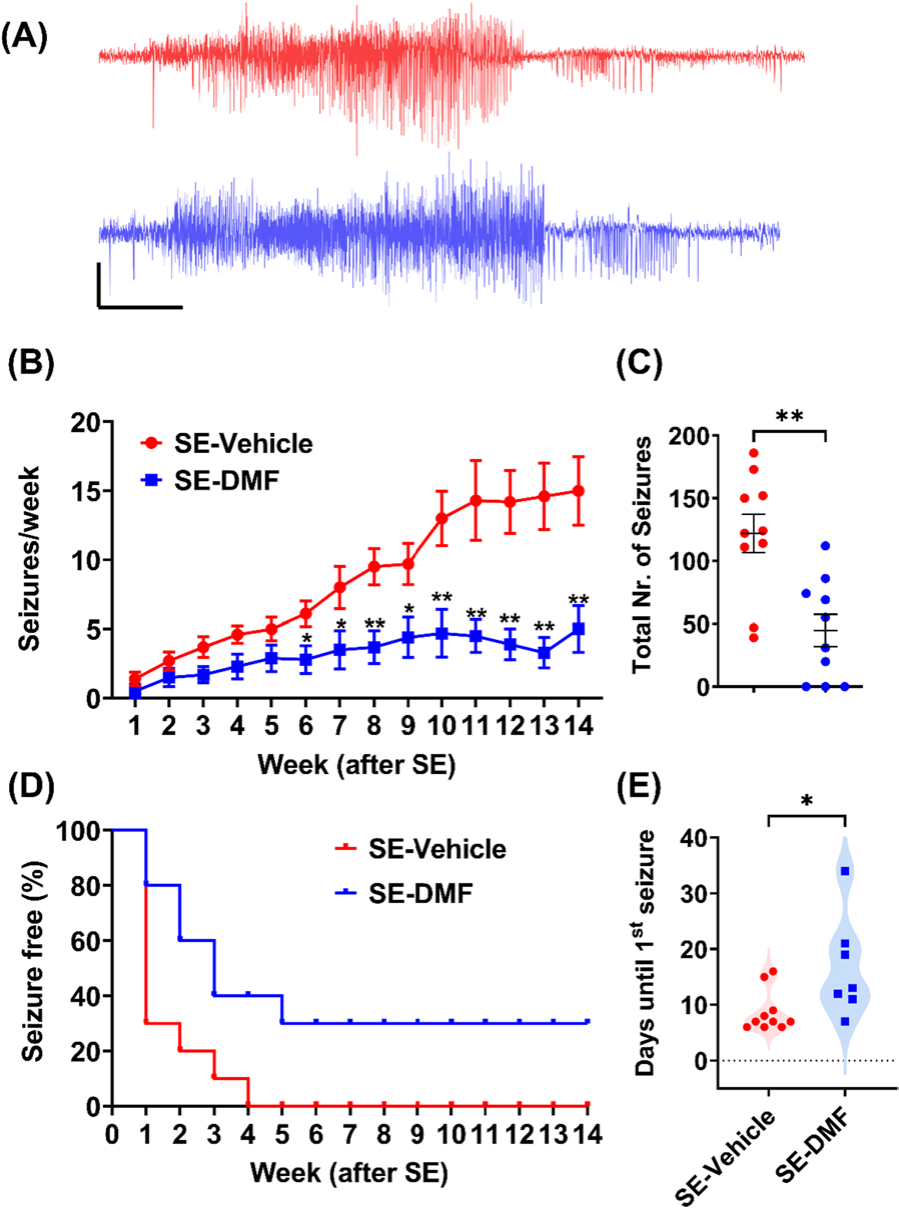
DMF suppresses the development of epilepsy after SE. **A** Representative seizures from two animals after KA-SE treated with either vehicle (red) or DMF (blue), scale bar, 10  s, and 0.5 mV. **B** Seizure frequency (per week, mean ± SEM) for animals treated with vehicle (n = 10 animals) or DMF (200 mg/kg/day for 7 days starting post-SE; n = 10 animals). Data were analyzed using a generalized log-linear mixed model followed by the Sidak post-hoc test. Time effect: P < 0.0001, F (13, 234) = 16.33; Treatment effect: P = 0.001, F (1, 18) = 15.00, Treatment × Time interaction effect: P < 0.0001, F (13, 234) = 6.228. * < 0.05, ** < 0.01 for comparison between groups in each time point. **C** Cumulative absolute number of seizures in animals in B. P = 0.0011, Student’s unpaired t-test. **D** Kaplan-Meier curve for the seizure-free effect of vehicle- or DMF-treated animals. **E** Latency period (days after SE until emergence of the 1st seizure) in vehicle- or DMF-treated animals. P = 0.0221, Student’s unpaired t-test

This study also determined whether the DMF-associated increase in the expression of Nrf2 and neuroprotection might translate into an anti-epileptogenic effect. We performed continuous video-ECoG recordings from rats subjected to 2 h of KA-SE and treated with vehicle or DMF. The ECoG data were analyzed for spontaneously recurring convulsive seizures, and all seizures were manually verified. Representative EEG traces from both vehicle- and DMF-treated rats are shown in Fig. 4A. The seizure frequency in vehicle-treated rats progressively increased after SE. Treatment with DMF for 1 week after SE significantly decreased seizure frequency in treated rats (Fig. 4B, F (13, 234) = 16.33, p < 0.0001, generalized log-linear mixed model). Moreover, DMF-treated rats experienced significantly fewer seizures than vehicle-treated rats (Fig. 4C, p = 0.001, unpaired t-test).

Interestingly, 10/10 vehicle-treated animals developed spontaneous seizures within 4 weeks after SE. In contrast, of 3/10 animals in the DMF-treated group remained seizure-free for the duration of the 14-week monitoring period post-SE (Fig. 4D). A log-rank test was calculated to test if DMF treatment can affect the distribution of time for seizure occurrence in all animals. Indeed, the distribution of time for seizure occurrence in the DMF treatment group significantly differs from the vehicle treated animals (p = 0.0051). Finally, the latency period (i.e., the time from SE onset until the emergence of the first spontaneous seizure) was significantly increased following DMF treatment (Median, Veh = 7 vs. DMF = 13 days, Fig. 4E).

### DMF reverses SE-induced behavioral changes in the open field and elevated plus-maze tests

**Fig. 5:**
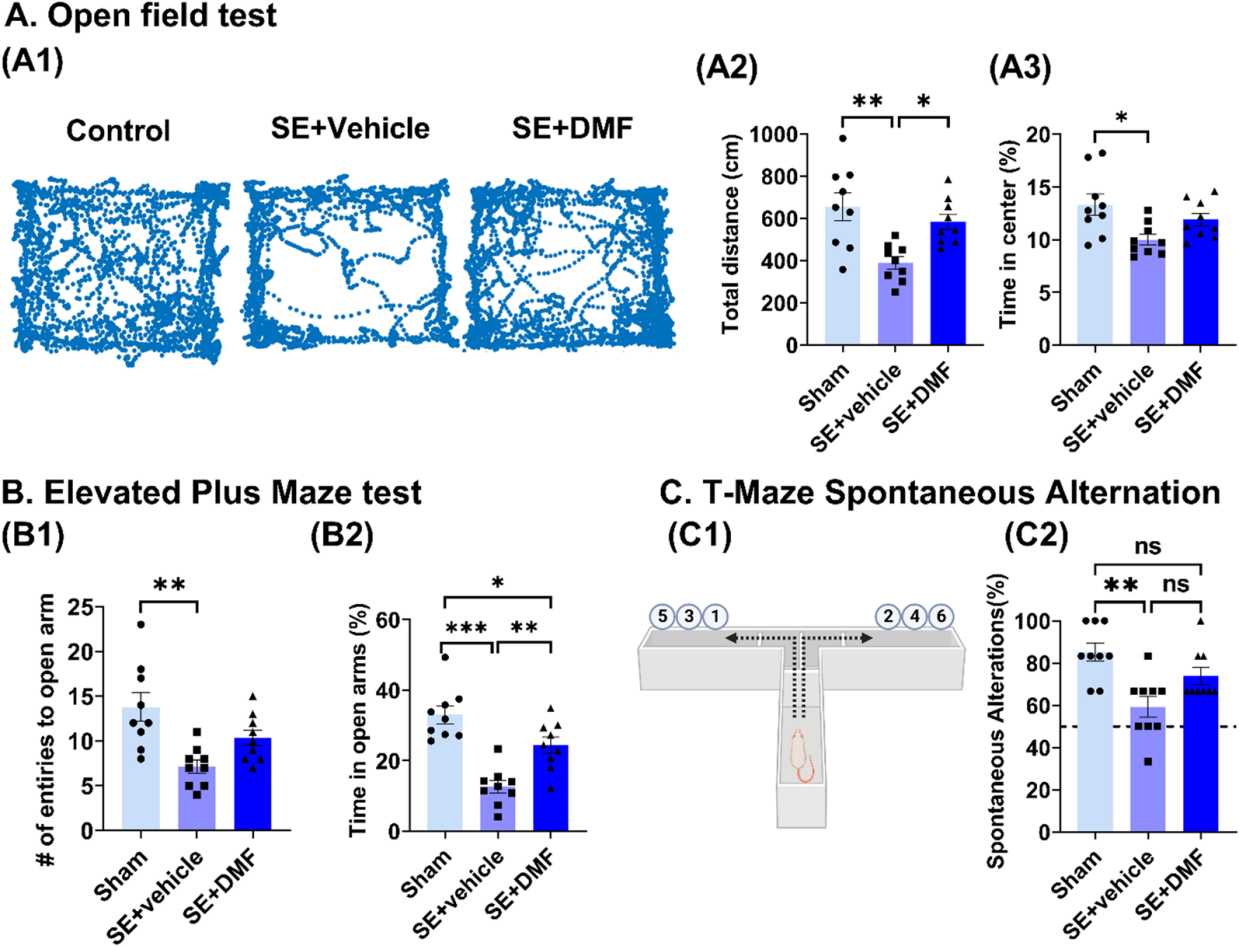
DMF reverses SE-associated behavioral changes in rats. Behavioral analysis of sham and KA-SE rats treated with vehicle or DMF in the open field (**A**), elevated plus-maze (**B**), and T-maze spontaneous alteration tests. **A1** Representative path of locomotion illustrating average activity in each group. **A2**, **A3** Distance traveled (cm) in the whole arena (**A2**) and percentage of time spent in the center (**A3**) during the 5-min testing sessions. **B1** Number of entries into the open arms of the EPM for sham animals and rats subjected to KA-SE and treated with vehicle or DMF. **B2** Time (%) spent in the open arms by animals from **B1**. **C1** Schematic drawing depicting the experimental setup used in the T-Maze spontaneous alteration T-maze paradigm. **C2** Spontaneous alternation rate in sham-operated animals and animals subjected to KA-SE and treated with vehicle or DMF. The number of animals for each group was n = 9. Data are expressed as the mean ± SEM. *P < 0.05, **P < 0.01, and ***P < 0.001 after one-way ANOVA followed by Tukey’s post hoc test

As shown in Fig. 5, a 7-day treatment with DMF beginning shortly after attenuation of SE resulted in normalization of behavior as assessed in the open field (OF; Fig. 5A) and elevated plus-maze tests (EPM; Fig. 5B), respectively. As noted in (Fig. 5A1), DMF-treated SE rats spent more time in the center of the arena and traveled longer distances than vehicle-treated rats (Fig. 5A2, A3). However, these outcomes were not statistically different from those of sham rats. As shown in (Fig. 5B), DMF-treated rats displayed more entries into the open arm of the EPM and spent more time in the open arm than vehicle-treated animals (Fig. 5B1, 5B2).

To evaluate memory function, we used the Spontaneous Alternation T-Maze test (Fig. 5C). Rats treated with vehicle after SE showed a significant decrease in the percentage of spontaneous alterations compared to sham animals, suggesting that epileptic animals exhibited impaired spatial memory (one-way ANOVA, F (2,24) = 8.580; p = 0.0015; Sham vs. SE + vehicle: p = 0.0011; Fig. 5C2). In DMF-treated animals, there was a slight increase in the percentage of spontaneous alterations compared to vehicle-treated animals (74% vs. 59%, respectively); however, this difference was not statistically significant (p = 0.0666). Importantly, there was no significant difference between sham-operated and DMF-treated animals (p = 0.2012), suggesting that DMF treatment shortly after SE can prevent epilepsy-induced memory dysfunction.

### DMF reduces symptomatic seizures in rats with chronic epilepsy

**Fig. 6:**
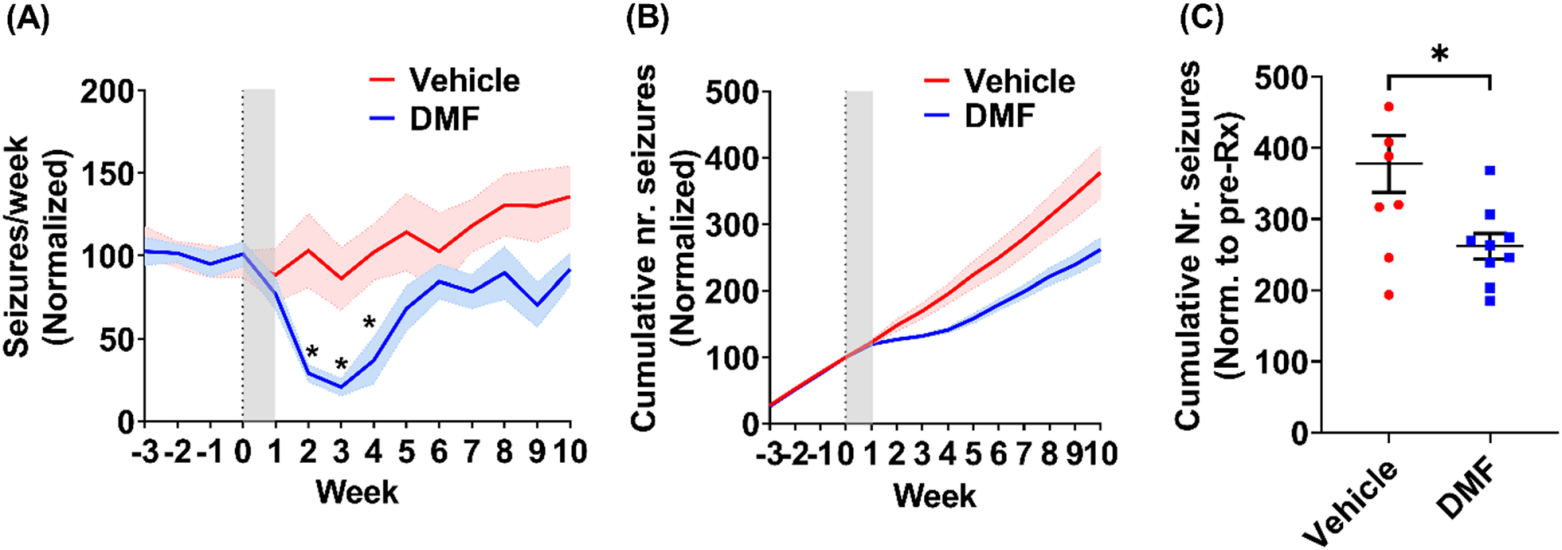
DMF reduces symptomatic seizures in rats with chronic epileptic animals. **A** Normalized seizure frequency (per week, mean ± SEM) in epileptic animals treated with either DMF (200 mg/kg/day for 7 days (shaded area), blue; n = 9 animals) or vehicle (equivalent doses of DMF; n = 9). Data were analyzed using a generalized log-linear mixed model followed by the Sidak post-hoc test. Time effect: P < 0.0001, F (13, 208) = 4.514; Treatment effect: P = 0.018, F (1, 16) = 6.947, Treatment × Time interaction effect: P = 0.0009, F (13, 208) = 2.833. * < 0.05 for comparison between groups at each time point. **B** Normalized cumulative number of seizures in animals in **A**. The number of seizures was normalized to the average number before treatment. **C** Total number of seizures at week 10 post-treatment of animals in a, normalized to baseline = 0.018, Student’s unpaired t-test

Finally, we investigated the therapeutic potential of DMF for chronic epilepsy. Rats were first subjected to KA-SE, and 10–12 weeks later, wireless electrocorticography (ECoG) transmitters were implanted to monitor the development of spontaneous seizures. We then recorded baseline ECoG activity for 3 weeks. Rats with confirmed epilepsy were randomized to receive treatment with either vehicle or DMF (200 mg/kg/day for 7 days; n = 9 rats/group). Video-ECoG recordings were continued during the treatment period for an additional 10–12 weeks. We found that treatment with DMF significantly reduced the normalized seizure frequency compared to that in vehicle-treated animals (generalized log-linear mixed model on weeks 1–10, treatment group × week interaction effect: F (8, 59) = 20.66, P < 0.001; Fig. 6A). Furthermore, the normalized cumulative number of seizures post-treatment was significantly lower in the DMF group than that in the vehicle control group (Fig. 6B, C, P = 0.0235, Student’s t-test).

## Discussion

In the present investigation, we demonstrated that early and late treatment with the Nrf2 activator DMF is disease-modifying in the post-SE rat model of acquired epilepsy. One week of DMF treatment beginning two hours after SE onset activated Nrf2-associated genes, enhanced neuroprotection in the hippocampus, attenuated epileptogenesis progression, and reversed SE-induced behavioral changes in the open field and elevated plus maze. Moreover, DMF treatment in rats with established epilepsy was associated with both an acute symptomatic improvement in seizure control and a prolonged reduction in seizure burden that extended well beyond the period when DMF was expected to be eliminated from the circulation. Collectively, these findings suggest that DMF, an Nrf2 activator, can modify the course of acquired epilepsy in a post-SE rat model.

We have previously shown that the protective response of Nrf2 signaling is endogenously activated in response to KA-induced SE [38] in a brain region-specific manner, with antioxidant systems being upregulated to a greater degree in the hippocampus than in the cortex. However, this study is the first to identify that exogenous treatment with DMF increases the activation of the Nrf2 pathway to higher levels than SE alone, which may explain the enhanced neuroprotection in the hippocampus of DMF-treated animals. These data suggest that the antioxidant capacity induced by SE alone is not sufficient for neuroprotection following SE. However, augmenting the natural antioxidant mechanisms of cells with Nrf2 activators represents a possible neuroprotective strategy. A previous study with a different Nrf2 activator, omaveloxolone, demonstrated similar effects on neuroprotection and subsequent epileptogenesis in rats following SE [34], supporting the hypothesis that Nrf2 activation is a possible therapeutic target for neuroprotection following neurological insults.

The kainic acid-induced status epilepticus (KA-SE) model is a widely used, well-validated model of temporal lobe epilepsy [42]. Neuronal damage across limbic regions, including the hippocampus, has been extensively characterized following KA-SE [35], and there is a mass of human and animal data supporting this following a brain insult. For example, there is evidence in humans that following SE (using longitudinal MRI), there is progressive hippocampal atrophy, which continues beyond 6 months [43]. The damage in these brain regions has been implicated in the development of epilepsy in these models. Hence, we hypothesized that DMF’s antiepileptogenic effects were mediated, in part, through neuroprotection in limbic brain structures. Therefore, we have analyzed neuronal cell density in CA1 and CA3 subregions of the hippocampus of DMF therapy vs. vehicle treated rats. Moreover, in our recent work [38], we found that Nrf2 expression following KA-SE model was increased predominantly in neurons of the CA1 and CA3 regions of the hippocampus, and no significant increase was detected in the cortex.

Prolonged seizures and SE initiate inflammatory responses, resulting in the production of reactive oxygen species (ROS), which contribute to neuronal damage [44–46]. Together, unresolved neuroinflammation and oxidative stress contribute to the development of epileptogenesis and network hyperexcitability [47–49].

In a recent study, Lucchi et al.[50] have demonstrated that human microglia have the capability to produce allopregnanolone, a neurosteroid that is released in higher amounts in response to oxidative stress, potentially contributing to microglial survival. Using the human microglial clone 3 (HMC3) cell line, it was found that in response to rotenone, an inhibitor of the mitochondrial complex I which produces oxidative damage, ROS levels were increased and the microglia viability was significantly reduced. Interestingly, these changes were accompanied by the decreased expression of multiple neurosteroids; whereas allopregnanolone levels exhibited a significant increase.

Moreover, Costa et al. [51] studied the therapeutic effect of trilostane, repeatedly reported to increase allopregnanolone levels in the brain. Despite not affecting the onset or duration of kainic acid-induced SE, trilostane exhibited a delayed onset of spontaneous seizures and reduced microglia activation. Repeated trilostane injections elevated allopregnanolone levels significantly, with a subsequent return to baseline after drug cessation, indicating allopregnanolone-associated protracted effects on epileptogenesis.

We found that the antioxidant response genes NQO1 and HO-1 were activated in response to DMF treatment following SE. These genes are critical for maintaining intracellular redox homeostasis and regulating inflammatory processes [52], suggesting that Nrf2 regulation of immune responses may explain both the anti-epileptogenic and acute anti-seizure effects observed with DMF. Surprisingly, the therapeutic effects of DMF continued well after elimination of the circulating drug, suggesting that an early and acute period of Nrf2 activation may be sufficient to alter disease processes after injury. However, the rebound effect observed 3 weeks after DMF treatment in rats with chronic seizures suggests that periodic treatment may be necessary to sustain the Nrf2-mediated benefits. Notably, we did not conduct an extended time course of Nrf2 activation following DMF treatment, which may be necessary to determine the duration of the treatment effects.

DMF treatment reversed the behavioral changes induced by SE, as assessed by the open field and elevated plus maze tests. Notably, DMF-treated SE rats spent more time in the center and traveled a greater distance in the open-field test. Similarly, rats displayed an increase in the number of entries and spent a greater amount of time in the open arm in the elevated plus maze test. On the surface, these findings suggest that SE is associated with an anxiolytic effect; an effect that is debated in the literature [53–55]. However, long-term behavioral changes associated with SE are strongly influenced by extrinsic factors, such as SE duration, epilepsy onset and severity, and age, all of which may explain the differences in anxiety-like behavior reported in the literature [53–55]. However, these findings could also be explained by increased hyperlocomotion, and the increased exploratory behavior observed in both the open field and elevated plus-maze tests may be a result of increased ambulation rather than decreased anxiety. What is clear is that DMF reverses the behavior observed in vehicle-treated post-SE rats. Given the neuroprotective effects observed in the hippocampus, it is perhaps not surprising that DMF can reverse SE-induced behavioral effects in the open field and elevated plus maze tests. Although the interpretation of these findings is limited, they support further investigations designed to assess whether DMF can mitigate the cognitive and behavioral comorbidities associated with SE and other brain insults.

Our study is not the first to evaluate the role of oxidative stress and Nrf2 activation in seizures and epilepsy. For example, the Nrf2 pathway is activated in the brains of amygdala-kindled rats [27], and further increases in Nrf2 expression via pharmacological or genetic approaches have been associated with anti-seizure and neuroprotective properties in pentylenetetrazol (PTZ)-kindled rats [56] and pilocarpine-induced SE in mice [57]. However, it is important to note that the administration of DMF before PTZ in these studies suggested that Nrf2-mediated protection was attributed to insult modification and not to disease modification. None of these studies evaluated the disease-modifying potential of DMF after a kindling stimulus or after the establishment of SE. Thus, this study is the first to evaluate the true disease-modifying potential of DMF in an etiological animal model of epileptogenesis. However, previous work with a different Nrf2 activator, omaveloxolone, has demonstrated both neuroprotective and disease-modifying properties in post-SE rats. Unlike DMF, the efficacy of omaveloxolone may be limited by its low tolerability; that is, weight loss was recorded at neuroprotective and disease-modifying doses [34]. In contrast to omaveloxolone, DMF is well tolerated at repeated doses ranging from 50 to 500 mg/kg [58], supporting its favorable safety profile in patients.

Few clinical trials have investigated the use of antioxidants in the treatment of epilepsy. Results with vitamin E, tirilazad, N-acetylcysteine, and ebselen [59–61] were mixed; however, these studies focused on the symptomatic treatment of epilepsy and were not designed to detect a disease-modifying effect. Importantly, therapy with antioxidants may need to be administered early and for a prolonged duration in chronic insidious neurologic diseases, such as epilepsy, to achieve an appreciable clinical benefit. Another factor limiting the efficacy of antioxidants is their poor penetration across the blood–brain barrier. DMF is ideally suited as a clinical candidate for the prevention and modification of insult-induced epilepsy because it is selective for Nrf2, orally bioavailable, and well-tolerated at repeated doses [58]. Clinical studies and post-marketing experiences further suggest that DMF is generally well-tolerated, with an acceptable safety profile [62]. Thus, targeting the Nrf2 pathway with the small-molecule activator DMF presents an attractive opportunity, because the target is an intrinsic cellular pathway designed to be dynamically modulated. While 30% of animals treated with DMF remained seizure-free in our study (vs. 0% in VEH animals), we envision that adjunctive DMF therapy to conventional antiseizure medicine therapy may provide better seizure control than ASM alone. Thus, given the fact that DMF is already approved, our current efforts are aimed at obtaining strong in vivo data in an etiologically relevant model of temporal lobe epilepsy that supports the central hypothesis that DMF treatment is disease modifying. This evidence can then serve to inform a future clinical trial.

## Conclusions

Overall, the effects observed following DMF treatment suggest that Nrf2 is a promising therapeutic target for preventing or attenuating epileptogenesis and associated comorbidities of epilepsy. DMF increased the expression of the downstream Nrf2 genes NQO1 and HO-1, which is consistent with its known effects on the Nrf2 pathway and provides a potential mechanism by which it could exert its therapeutic effects in preclinical models of epilepsy. However, further research is needed to fully understand the relationship between DMF and the Nrf2 pathway and its potential therapeutic effects in epilepsy and other neurological disorders. Regardless, the findings reported herein are important, as they support further evaluation of DMF as a potential first-in-class disease-modifying agent for the prevention and modification of epilepsy and attendant comorbidities of epilepsy following an insult to the brain, such as traumatic brain injury, stroke, brain tumor, infection, neurodegeneration, or prolonged status epilepticus.

### Supplementary Information

**Additional file 1: Figure S1.** **a** Total KA doses administered to rats in the vehicle and DMF treatment groups (about Fig. 4). No difference was detected between the groups. **a** The duration of SE as recorded by ECoG device in animals in (**a**). Data are displayed as the mean ± SEM. **A** P = 0.6435; **B** P = 0.3408) analyzed by students’ unpaired t-test. **Figure S2.** Protein expression of Nrf2-related genes in the cortex. **a** Representative western blots of the Nrf2 downstream genes NQO1, HO-1, and GCLC-1 in the cortex of sham rats (n = 6), as well as rats subjected to KA-SE and treated with either Vehicle (n = 6) or DMF (n = 6). **b**–**d** Quantification of western blot results in (**A**). Data are displayed as the mean ± SEM, analyzed by one-way ANOVA followed by Tukey posthoc test. *P < 0.05; **P < 0.01; *** < 0.001. **Figure S3.** Protein expression of Nrf2-related genes in the hippocampus. **a** Representative western blots of the Nrf2 downstream genes NQO1, HO-1, and GCLC-1 in the hippocampus of sham rats (n = 6), as well as rats subjected to KA-SE and treated with either Vehicle (n = 6) or DMF (n = 6). **b**–**d** Quantification of western blot results in (A). Data are displayed as the mean ± SEM, analyzed by one-way ANOVA followed by Tukey posthoc test. **P < 0.01.

## Data Availability

All data generated or analyzed during this study are included in this published article and its supplementary information files.

## Abbreviations

ASMs: Antiseizure medications
DMF: Dimethyl fumarate
DRE: Drug-resistant epilepsy
ECoG: Electrocorticography
EPM: Elevated plus maze
GAPDH: Glyceraldehyde-3-phosphate dehydrogenase
GCLC1: Glutamate-cysteine ligase catalytic subunit
HO-1: Heme-oxygenase 1
KA: Kainic acid
NQO1: NAD(P)H: quinone oxidoreductase-1
Nrf2: Nuclear factor-erythroid 2-related factor 2
OF: Open-field
PFA: Paraformaldehyde
SE: Status epilepticus
SRS: Spontaneous recurrent seizures
TLE: Temporal lobe epilepsy

## References

[1] Zeng LH, Rensing NR, Wong M (2009). The mammalian target of rapamycin signaling pathway mediates epileptogenesis in a model of temporal lobe epilepsy. J Neurosci.

[2] Devinsky O, Vezzani A, O'Brien TJ, Jette N, Scheffer IE, de Curtis M (2018). Epilepsy. Nat Rev Dis Primers.

[3] Devinsky O, Spruill T, Thurman D, Friedman D (2016). Recognizing and preventing epilepsy-related mortality: a call for action. Neurology.

[4] Manolis TA, Manolis AA, Melita H, Manolis AS (2019). Sudden unexpected death in epilepsy: the neuro-cardio-respiratory connection. Seizure.

[5] Chen Z, Brodie MJ, Liew D, Kwan P (2018). Treatment outcomes in patients with newly diagnosed epilepsy treated with established and new antiepileptic drugs: a 30-year longitudinal cohort study. JAMA Neurol.

[6] Golyala A, Kwan P (2017). Drug development for refractory epilepsy: the past 25 years and beyond. Seizure.

[7] Engel T, Martinez-Villarreal J, Henke C, Jimenez-Mateos EM, Sanz-Rodriguez A, Alves M (2017). Spatiotemporal progression of ubiquitin-proteasome system inhibition after status epilepticus suggests protective adaptation against hippocampal injury. Mol Neurodegener.

[8] Laxer KD, Trinka E, Hirsch LJ, Cendes F, Langfitt J, Delanty N (2014). The consequences of refractory epilepsy and its treatment. Epilepsy Behav.

[9] Perucca P, Scheffer IE, Kiley M (2018). The management of epilepsy in children and adults. Med J Aust.

[10] Fattorusso A, Matricardi S, Mencaroni E, Dell'Isola GB, Di Cara G, Striano P (2021). The pharmacoresistant epilepsy: an overview on existant and new emerging therapies. Front Neurol.

[11] Dichter MA (2006). Models of epileptogenesis in adult animals available for antiepileptogenesis drug screening. Epilepsy Res.

[12] Löscher W, Brandt C (2010). Prevention or modification of epileptogenesis after brain insults: experimental approaches and translational research. Pharmacol Rev.

[13] Peixoto-Santos JE, Velasco TR, Carlotti CG, Assirati JA, Rezende G, Kobow K (2020). Histological correlates of hippocampal magnetization transfer images in drug-resistant temporal lobe epilepsy patients. Neuroimage Clin.

[14] Thom M (2014). Review: hippocampal sclerosis in epilepsy: a neuropathology review. Neuropathol Appl Neurobiol.

[15] Bandopadhyay R, Liu JY, Sisodiya SM, Thom M (2014). A comparative study of the dentate gyrus in hippocampal sclerosis in epilepsy and dementia. Neuropathol Appl Neurobiol.

[16] Kwon HS, Koh SH (2020). Neuroinflammation in neurodegenerative disorders: the roles of microglia and astrocytes. Transl Neurodegener.

[17] Liddell JR (2017). Are astrocytes the predominant cell type for activation of Nrf2 in aging and neurodegeneration?. Antioxidants (Basel)..

[18] Emerit J, Edeas M, Bricaire F (2004). Neurodegenerative diseases and oxidative stress. Biomed Pharmacother.

[19] Barnham KJ, Masters CL, Bush AI (2004). Neurodegenerative diseases and oxidative stress. Nat Rev Drug Discov.

[20] Carmona-Aparicio L, Perez-Cruz C, Zavala-Tecuapetla C, Granados-Rojas L, Rivera-Espinosa L, Montesinos-Correa H (2015). Overview of Nrf2 as therapeutic target in epilepsy. Int J Mol Sci.

[21] Miller DM, Wang JA, Buchanan AK, Hall ED (2014). Temporal and spatial dynamics of nrf2-antioxidant response elements mediated gene targets in cortex and hippocampus after controlled cortical impact traumatic brain injury in mice. J Neurotrauma.

[22] Cuadrado A, Rojo AI, Wells G, Hayes JD, Cousin SP, Rumsey WL (2019). Therapeutic targeting of the NRF2 and KEAP1 partnership in chronic diseases. Nat Rev Drug Discov.

[23] da Costa RM, Rodrigues D, Pereira CA, Silva JF, Alves JV, Lobato NS (2019). Nrf2 as a potential mediator of cardiovascular risk in metabolic diseases. Front Pharmacol.

[24] Sandouka S, Shekh-Ahmad T (2021). Induction of the Nrf2 pathway by sulforaphane is neuroprotective in a rat temporal lobe epilepsy model. Antioxidants (Basel)..

[25] Tu J, Zhang X, Zhu Y, Dai Y, Li N, Yang F (2015). Cell-permeable peptide targeting the Nrf2-Keap1 interaction: a potential novel therapy for global cerebral ischemia. J Neurosci.

[26] Kraft AD, Lee JM, Johnson DA, Kan YW, Johnson JA (2006). Neuronal sensitivity to kainic acid is dependent on the Nrf2-mediated actions of the antioxidant response element. J Neurochem.

[27] Wang W, Wang WP, Zhang GL, Wu YF, Xie T, Kan MC (2013). Activation of Nrf2-ARE signal pathway in hippocampus of amygdala kindling rats. Neurosci Lett.

[28] Saidu NEB, Kavian N, Leroy K, Jacob C, Nicco C, Batteux F (2019). Dimethyl fumarate, a two-edged drug: current status and future directions. Med Res Rev.

[29] Al-Jaderi Z, Maghazachi AA (2016). Utilization of dimethyl fumarate and related molecules for treatment of multiple sclerosis, cancer, and other diseases. Front Immunol.

[30] Zhao X, Sun G, Zhang J, Ting SM, Gonzales N, Aronowski J (2015). Dimethyl fumarate protects brain from damage produced by intracerebral hemorrhage by mechanism involving Nrf2. Stroke.

[31] Scannevin RH, Chollate S, Jung MY, Shackett M, Patel H, Bista P (2012). Fumarates promote cytoprotection of central nervous system cells against oxidative stress via the nuclear factor (erythroid-derived 2)-like 2 pathway. J Pharmacol Exp Ther.

[32] Brennan MS, Matos MF, Li B, Hronowski X, Gao B, Juhasz P (2015). Dimethyl fumarate and monoethyl fumarate exhibit differential effects on KEAP1, NRF2 activation, and glutathione depletion in vitro. PLoS ONE.

[33] Hellier JL, Patrylo PR, Buckmaster PS, Dudek FE (1998). Recurrent spontaneous motor seizures after repeated low-dose systemic treatment with kainate: assessment of a rat model of temporal lobe epilepsy. Epilepsy Res.

[34] Shekh-Ahmad T, Eckel R, Dayalan Naidu S, Higgins M, Yamamoto M, Dinkova-Kostova AT (2018). KEAP1 inhibition is neuroprotective and suppresses the development of epilepsy. Brain.

[35] Ben-Ari Y (1985). Limbic seizure and brain damage produced by kainic acid: mechanisms and relevance to human temporal lobe epilepsy. Neuroscience.

[36] Shekh-Ahmad T, Lieb A, Kovac S, Gola L, Christian Wigley W, Abramov AY (2019). Combination antioxidant therapy prevents epileptogenesis and modifies chronic epilepsy. Redox Biol.

[37] Livak KJ, Schmittgen TD (2001). Analysis of relative gene expression data using real-time quantitative PCR and the 2(-Delta Delta C(T)) Method. Methods.

[38] Sandouka S, Saadi A, Singh PK, Olowe R, Shekh-Ahmad T (2023). Nrf2 is predominantly expressed in hippocampal neurons in a rat model of temporal lobe epilepsy. Cell Biosci.

[39] Hopkins KJ, Wang G, Schmued LC (2000). Temporal progression of kainic acid induced neuronal and myelin degeneration in the rat forebrain. Brain Res.

[40] Jupp B, Williams J, Binns D, Hicks RJ, Cardamone L, Jones N (2012). Hypometabolism precedes limbic atrophy and spontaneous recurrent seizures in a rat model of TLE. Epilepsia.

[41] Maia GH, Quesado JL, Soares JI, Carmo do JM, Andrade PA, Andrade JP (2014). Loss of hippocampal neurons after kainate treatment correlates with behavioral deficits. PLoS ONE..

[42] Bertoglio D, Amhaoul H, Van Eetveldt A, Houbrechts R, Van De Vijver S, Ali I (2017). Kainic acid-induced post-status epilepticus models of temporal lobe epilepsy with diverging seizure phenotype and neuropathology. Front Neurol.

[43] Gong G, Shi F, Concha L, Beaulieu C, Gross DW (2008). Insights into the sequence of structural consequences of convulsive status epilepticus: a longitudinal MRI study. Epilepsia.

[44] Liu J, Wang A, Li L, Huang Y, Xue P, Hao A (2010). Oxidative stress mediates hippocampal neuron death in rats after lithium-pilocarpine-induced status epilepticus. Seizure.

[45] Wang A, Si Z, Xue P, Li X, Liu J (2019). Tacrolimus protects hippocampal neurons of rats with status epilepticus through suppressing oxidative stress and inhibiting mitochondrial pathway of apoptosis. Brain Res.

[46] Lin TK, Chen SD, Lin KJ, Chuang YC (2020). Seizure-induced oxidative stress in status epilepticus: is antioxidant beneficial?. Antioxidants (Basel)..

[47] Soehnlein O, Lindbom L (2010). Phagocyte partnership during the onset and resolution of inflammation. Nat Rev Immunol.

[48] Arena A, Zimmer TS, van Scheppingen J, Korotkov A, Anink JJ, Muhlebner A (2019). Oxidative stress and inflammation in a spectrum of epileptogenic cortical malformations: molecular insights into their interdependence. Brain Pathol.

[49] Saha S, Buttari B, Panieri E, Profumo E, Saso L (2020). An overview of Nrf2 signaling pathway and its role in inflammation. Molecules..

[50] Lucchi C, Codeluppi A, Filaferro M, Vitale G, Rustichelli C, Avallone R (2023). Human microglia synthesize neurosteroids to cope with rotenone-induced oxidative stress. Antioxidants (Basel)..

[51] Costa AM, Gol M, Lucchi C, Biagini G (2023). Antiepileptogenic effects of trilostane in the kainic acid model of temporal lobe epilepsy. Epilepsia.

[52] Wardyn JD, Ponsford AH, Sanderson CM (2015). Dissecting molecular cross-talk between Nrf2 and NF-kappaB response pathways. Biochem Soc Trans.

[53] Muller CJ, Groticke I, Bankstahl M, Loscher W (2009). Behavioral and cognitive alterations, spontaneous seizures, and neuropathology developing after a pilocarpine-induced status epilepticus in C57BL/6 mice. Exp Neurol.

[54] Castelhano AS, Cassane Gdos S, Scorza FA, Cysneiros RM (2013). Altered anxiety-related and abnormal social behaviors in rats exposed to early life seizures. Front Behav Neurosci.

[55] Aguilar BL, Malkova L, N'Gouemo P, Forcelli PA (2018). Genetically epilepsy-prone rats display anxiety-like behaviors and neuropsychiatric comorbidities of epilepsy. Front Neurol.

[56] Singh N, Vijayanti S, Saha L, Bhatia A, Banerjee D, Chakrabarti A (2018). Neuroprotective effect of Nrf2 activator dimethyl fumarate, on the hippocampal neurons in chemical kindling model in rat. Epilepsy Res.

[57] Mazzuferi M, Kumar G, van Eyll J, Danis B, Foerch P, Kaminski RM (2013). Nrf2 defense pathway: experimental evidence for its protective role in epilepsy. Ann Neurol.

[58] Clulow JA, Storck EM, Lanyon-Hogg T, Kalesh KA, Jones LH, Tate EW (2017). Competition-based, quantitative chemical proteomics in breast cancer cells identifies new target profiles for sulforaphane. Chem Commun (Camb).

[59] Raju GB, Behari M, Prasad K, Ahuja GK (1994). Randomized, double-blind, placebo-controlled, clinical trial of D-alpha-tocopherol (vitamin E) as add-on therapy in uncontrolled epilepsy. Epilepsia.

[60] Mehvari J, Motlagh FG, Najafi M, Ghazvini MR, Naeini AA, Zare M (2016). Effects of Vitamin E on seizure frequency, electroencephalogram findings, and oxidative stress status of refractory epileptic patients. Adv Biomed Res.

[61] Delanty N, Dichter MA (2000). Antioxidant therapy in neurologic disease. Arch Neurol.

[62] Carrasco-Pozo C, Tan KN, Borges K (2015). Sulforaphane is anticonvulsant and improves mitochondrial function. J Neurochem.

