# Supplementary material for: Repurposing dimethyl fumarate as an antiepileptogenic and disease-modifying treatment for drug-resistant epilepsy
Source: J Transl Med. 2023 Nov 8;21:796. doi: 10.1186/s12967-023-04695-2 (PMC10634153; doi:10.1186/s12967-023-04695-2)
Supplement: Supplementary file 1 — Additional file 1: Figure S1. a Total KA doses administered to rats in the vehicle and DMF treatment groups (about Fig. 4). No difference was detected between the groups. a The duration of SE as recorded by ECoG device in animals in (a). Data are displayed as the mean ± SEM. A P = 0.6435; B P = 0.3408) analyzed by students’ unpaired t-test. Figure S2. Protein expression of Nrf2-related genes in the cortex. a Representative western blots of the Nrf2 downstream genes NQO1, HO-1, and GCLC-1 in the cortex of sham rats (n = 6), as well as rats subjected to KA-SE and treated with either Vehicle (n = 6) or DMF (n = 6). b–d Quantification of western blot results in (A). Data are displayed as the mean ± SEM, analyzed by one-way ANOVA followed by Tukey posthoc test. *P < 0.05; **P < 0.01; *** < 0.001. Figure S3. Protein expression of Nrf2-related genes in the hippocampus. a Representative western blots of the Nrf2 downstream genes NQO1, HO-1, and GCLC-1 in the hippocampus of sham rats (n = 6), as well as rats subjected to KA-SE and treated with either Vehicle (n = 6) or DMF (n = 6). b–d Quantification of western blot results in (A). Data are displayed as the mean ± SEM, analyzed by one-way ANOVA followed by Tukey posthoc test. **P < 0.01. [file 12967_2023_4695_MOESM1_ESM.docx]

**Supporting Information**

**List of Supporting Information**

**Figure S1**

**Figure S2**

**Figure S3**


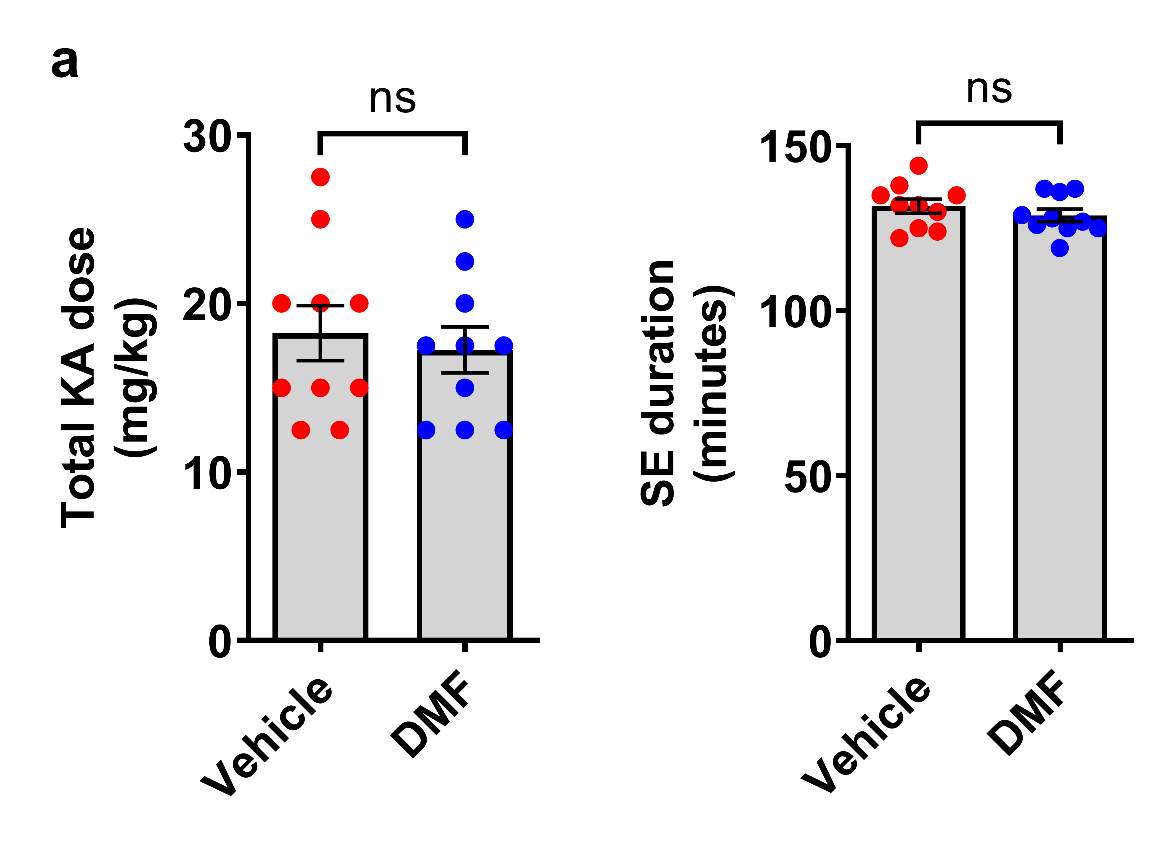


**Figure S1**

(a) Total KA doses administered to rats in the vehicle and DMF treatment groups (about Figure 4). No difference was detected between the groups.

(a) The duration of SE as recorded by ECoG device in animals in (a).

Data are displayed as the mean ± SEM. A: P=0.6435; B: P = 0.3408) analyzed by students’ unpaired t-test


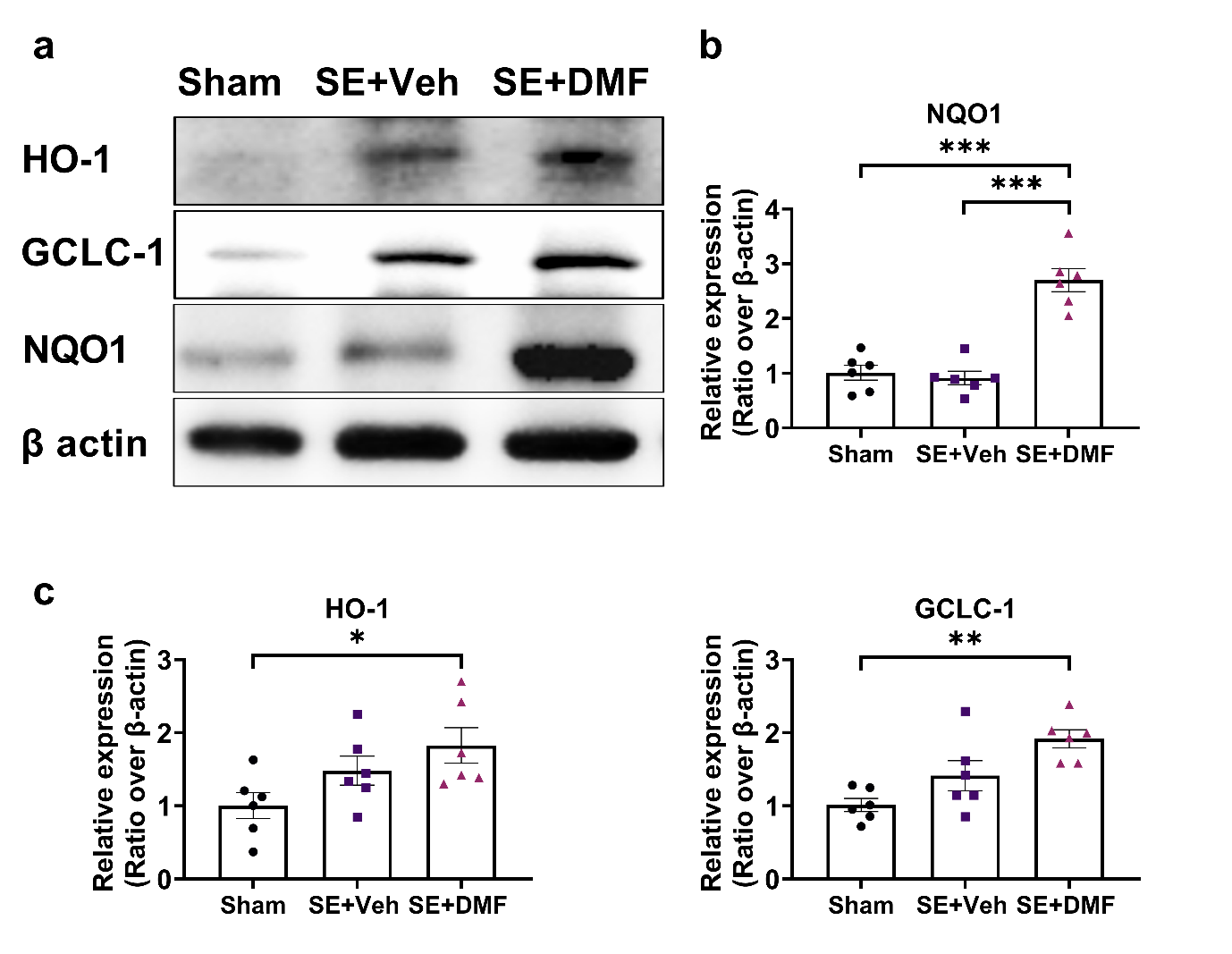


**Figure S2. Protein expression of Nrf2-related genes in the cortex**

(a) Representative western blots of the Nrf2 downstream genes NQO1, HO-1, and GCLC-1 in the cortex of sham rats (n = 6), as well as rats subjected to KA-SE and treated with either Vehicle (n = 6) or DMF (n = 6).

(b-d) Quantification of western blot results in (A).

Data are displayed as the mean ± SEM, analyzed by one-way ANOVA followed by Tukey posthoc test. * P < 0.05; ** P < 0.01; *** < 0.001.


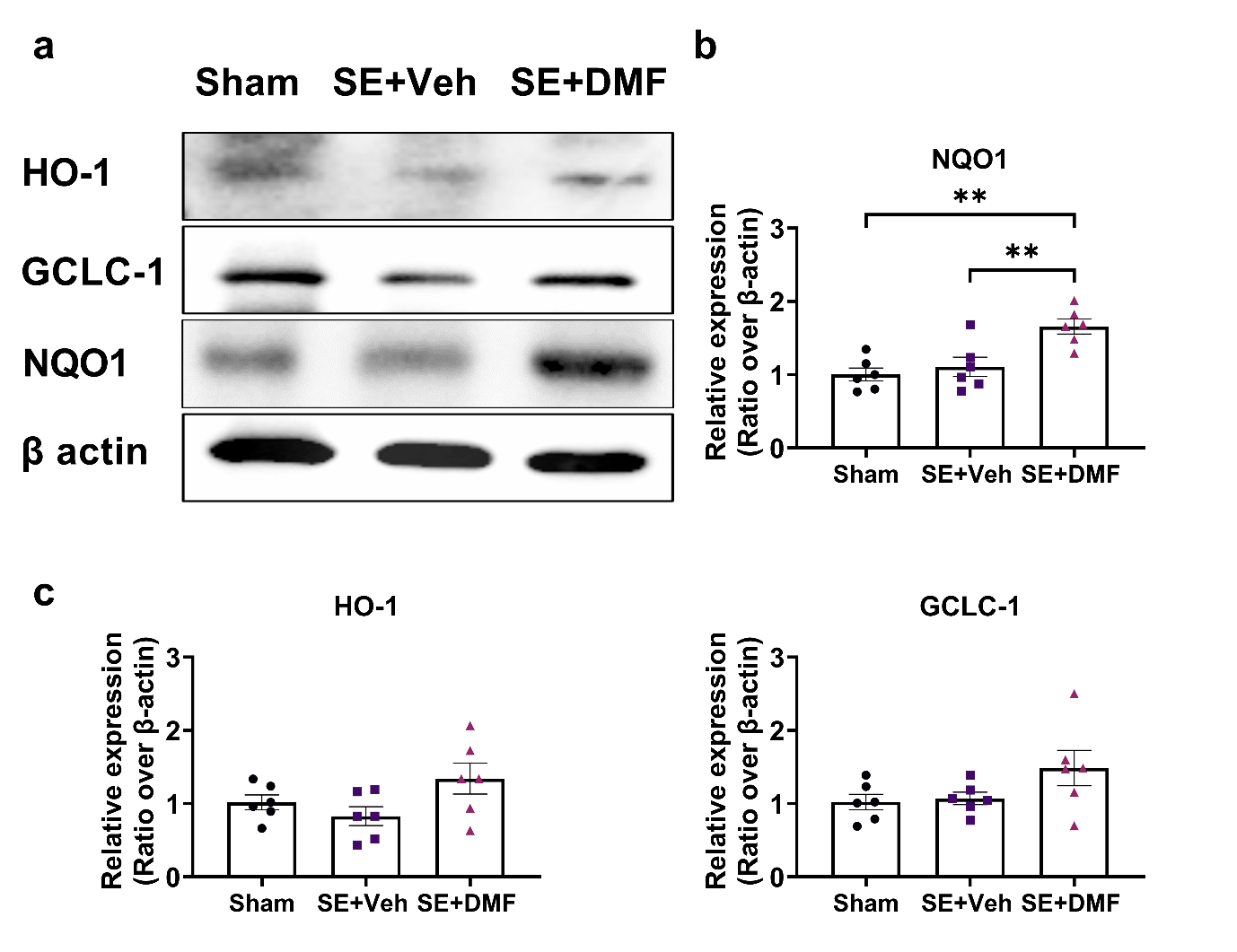


**Figure S3. Protein expression of Nrf2-related genes in the hippocampus.**

(a) Representative western blots of the Nrf2 downstream genes NQO1, HO-1, and GCLC-1 in the hippocampus of sham rats (n=6), as well as rats subjected to KA-SE and treated with either Vehicle (n=6) or DMF (n=6).

(b-d) Quantification of western blot results in (A).

Data are displayed as the mean ±SEM, analyzed by one-way ANOVA followed by Tukey posthoc test. ** P < 0.01.
